# Wearables and Smartphones for Tracking Modifiable Risk Factors in Metabolic Health: Protocol for a Scoping Review

**DOI:** 10.2196/59539

**Published:** 2024-11-28

**Authors:** Victoria Brügger, Tobias Kowatsch, Mia Jovanova

**Affiliations:** 1 School of Medicine University of St. Gallen St. Gallen Switzerland; 2 Institute for Implementation Science in Health Care University of Zurich Zurich Switzerland; 3 Department of Management, Technology and Economics ETH Zürich Zurich Switzerland

**Keywords:** wearable, smartphone, mHealth, metabolic disease, lifestyle, physiological, risk factor, mobile phone

## Abstract

**Background:**

Metabolic diseases, such as cardiovascular diseases and diabetes, contribute significantly to global mortality and disability. Wearable devices and smartphones are increasingly used to track and manage modifiable risk factors associated with metabolic diseases. However, no established guidelines exist on how to derive meaningful signals from these devices, often hampering cross-study comparisons.

**Objective:**

This study aims to systematically overview the current empirical literature on how wearables and smartphones are used to track modifiable (physiological and lifestyle) risk factors associated with metabolic diseases.

**Methods:**

We will conduct a scoping review to overview how wearable and smartphone-based studies measure modifiable risk factors related to metabolic diseases. We will search 5 databases (Scopus, Web of Science, PubMed, Cochrane Central Register of Controlled Trials, and SPORTDiscus) from 2019 to 2024, with search terms related to wearables, smartphones, and modifiable risk factors associated with metabolic diseases. Eligible studies will use smartphones or wearables (worn on the wrist, finger, arm, hip, and chest) to track physiological or lifestyle factors related to metabolic diseases. We will follow the reporting guideline standards from PRISMA-ScR (Preferred Reporting Items for Systematic Reviews and Meta-Analyses Extension for Scoping Reviews) and the JBI (Joanna Briggs Institute) guidance on scoping review methodology. Two reviewers will independently screen articles for inclusion and extract data using a standardized form. The findings will be synthesized and reported qualitatively and quantitatively.

**Results:**

Data collection is expected to begin in November 2024; data analysis in the first quarter of 2025; and submission to a peer-reviewed journal by the second quarter of 2025. We expect to identify the degree to which wearable and smartphone-based studies track modifiable risk factors collectively (versus in isolation), and the consistency and variation in how modifiable risk factors are measured across existing studies.

**Conclusions:**

Results are expected to inform more standardized guidelines on wearable and smartphone-based measurements, with the goal of aiding cross-study comparison. The final report is planned for submission to a peer-reviewed, indexed journal. This review is among the first to systematically overview the current landscape on how wearables and smartphones measure modifiable risk factors associated with metabolic diseases.

**International Registered Report Identifier (IRRID):**

PRR1-10.2196/59539

## Introduction

Noncommunicable diseases lead to 41 million deaths per year globally and are estimated to cost, on average, more than US $2 trillion per year [1,2]. A large portion of noncommunicable diseases–related burden is attributed to a growing prevalence of metabolic diseases, namely type 2 diabetes, hypertension, hyperlipidemia, obesity, and, more recently, nonalcoholic fatty liver disease [3,4]. Metabolic diseases are projected to increase significantly, with diabetes prevalence rates expected to double from 529 million in 2021 to 1.3 billion in 2050, and related expenditures projected to surpass US $1054 billion by 2045 [5].

A large body of research has shown that metabolic diseases, for example, type 2 diabetes, are influenced by a complex network of modifiable factors. These include lifestyle factors (ie, nutrition, physical activity, sleep, stress, and substance abuse) and physiological markers (ie, blood sugar, triglycerides, and high-density lipoprotein cholesterol) [6-10]. Following complex systems perspectives, modifiable factors interact [11,12] and together shape disease outcomes over time, with up to 70% of cardiovascular disease cases and mortality attributed to modifiable risk factors [8,9,12].

In parallel, wearables (ie, devices worn on the wrist, finger, arm, and chest) and smartphones are increasingly used to track modifiable risk factors in daily life, with improved precision and accuracy [13]. These digital devices offer key advantages over lab-based measurements, such as continuous and person-specific data collection in (near) real-time. For instance, smartwatches can track physical activity and sleep patterns in the natural environment with minimal burden [14]. Further, mobile apps can detect dietary patterns through image-based food recognition [15] and brief ecologic momentary assessments [16] in free-living conditions. These high-dimensional, longitudinal data enable continuous monitoring and can be used to trigger personalized lifestyle interventions, for example by personalizing recommendations on nutrition, sleep, and physical activity [14,16,17]. By enabling remote monitoring, wearables, and smartphones can potentially improve access to care and reduce the costs of metabolic disease management [18,19] versus standard lab-based clinical approaches [20].

While studies increasingly demonstrate the promise of wearables for tracking, preventing, and managing metabolic diseases [21], several key gaps remain in the newly evolving field of digital metabolic health. Here, we focus on two gaps. First, recent perspectives highlight the importance of tracking multiple, modifiable risk factors in parallel (vs a single risk factor in isolation) for a more comprehensive lens into an individual’s metabolic health profile [22]. However, the extent to which existing studies focus on multiple versus risk factors remains unclear. Second, alongside the proliferation of wearables and smartphones, there are concerns regarding data comparability, even when researchers aim to measure the same risk factors [23]. Specifically, there is growing evidence of incommensurability, with researchers employing different operationalizations and measures of the same risk factors (ie, physical inactivity), thus making direct cross-study comparisons challenging. This heterogeneity can make it difficult to directly track between-study effects [20] and present a barrier to building cumulative and generalizable knowledge [24]. Motivated by these gaps, it is essential to examine which modifiable risk factors are measured using wearables and smartphones and how recent work has begun to overview the use of different wearable technologies in cardiometabolic diseases [20] and the role of digital health technologies in metabolic disorders among older adults, more broadly [25]. However, to our knowledge, no studies have specifically focused on the landscape of modifiable risk factors. Thus, we aim to address the two questions, that are (1) Which modifiable risk factors are most often studied in wearable and smartphone-based metabolic health research? and (2) To what extent are measures of modifiable risk factors consistent across studies, particularly in measurement methods?

Gaining a comprehensive understanding of the current landscape of modifiable risk factors, tracked using wearables and smartphones, is crucial to inform more consistent measurement guidelines. Given the broad nature of the inquiry and the emerging status of the field, we deemed a scoping review the most appropriate method for investigating these research questions.

## Methods

### Overview

We will follow the 2020 JBI methodological guidance for scoping reviews [26,27], developed by the JBI Scoping Review Methodology Group. Accordingly, our scoping review is structured to follow seven stages, which consist of (1) title and research question, (2) identifying inclusion criteria, (3) identifying a search strategy, (4) evidence screening and selection, (5) data extraction, (6) data analysis, and (7) presentation of results. To guarantee adherence to reporting, we will also follow the PRISMA-ScR (Preferred Reporting Items for Systematic Reviews and Meta-Analyses Extension for Scoping Reviews) checklist [28].

### Stage 1: Title and Research Question

The title was developed based on the PCC (population, concept, and context) mnemonic [26], with a focus on the concept and context, namely wearables and smartphones for modifiable risk factors. The title does not explicitly mention the study population due to our broad inclusion criteria (eg, any adults). Motivated by gaps in previous work [20,29] and a lack of overview in the field, this scoping review aims to systematically describe (1) which modifiable risk factors are most often studied in wearable and smartphone-based metabolic health research, and (2) to what extent are measures of modifiable risk factors consistent across studies in terms of measurement methods.

### Stage 2: Inclusion and Exclusion Criteria

Eligible studies will be peer-reviewed, written in English, published in 2019 and after (to reflect the most current literature and build on previous work [30]); and will include a full-text version. Given our research aims, eligible studies will: (1) measure lifestyle and physiological factors relevant to metabolic health, use (2) smartphones or wearables worn on the wrist, finger, arm, and chest, and (3) analyze the generated wearables or smartphone data for outcome assessment related to metabolic health. Qualitative studies will be excluded. Building on previous work [30], we will include wearable devices that cost <€500 (US $570) per device hardware, consistent with price cut-offs in previous work [30]. We will include membership-based devices. The study population will include adults aged 18 years and older. We will use institutional journal subscriptions and interlibrary loan services to access studies, and in cases where no full text is available, authors will be contacted with a waiting period of 7 days before study exclusion. Textbox 1 presents the study’s inclusion and exclusion criteria.

**Study inclusion and exclusion criteria.**

**Inclusion Criteria**

- Peer-reviewed empirical study
- Published from 2019 to 2024
- The study involves wearables or smartphones for data collection
- The study focuses on modifiable risk factors in metabolic health or disease contexts (a minimum of one factor from Table S1 in Multimedia Appendix 1 is included)

**Exclusion Criteria**

- The article is not in English
- The publication type is not an original empirical article (ie, conference abstract, commentary, or letters to the editor)
- No quantitative analyses
- Wearable costs≥500€ (US $570)
- Study population age <18 years
- Primary research objectives and analyses do not involve wearable or smartphone-collected data, or the outcome is not related to metabolic health

### Stage 3: Search Strategy

Building on the most recent reviews in the wearable, digital health, and metabolic health domains [20,29], we identified key search terms capturing wearables or smartphones and prominent modifiable risk factors associated with metabolic diseases [6]. A full list of key search terms can be found in Table S2 in Multimedia Appendix 1. We will search the following 5 major databases, that are Scopus, Web of Science, PubMed, Cochrane Centralized Register of Controlled Trials, and SportDiscus. Identified studies will be imported into the systematic review tool Rayyan developed by the Qatar Computing Research Institute [31].

### Stage 4: Evidence Screening and Selection

Study evidence screening and selection will be performed based on the inclusion and exclusion criteria (see Textbox 1). We will first screen the title and abstract based on the predefined criteria, followed by screening the full text. This process will be conducted by 2 reviewers in parallel, and any discrepancies in study eligibility will be resolved by a third reviewer.

### Stage 5: Data Extraction

For each eligible study, we will extract information using a standardized data charting form. In total, 2 reviewers will manually chart the extracted information, and a third reviewer will resolve any discrepancies. The data charting form is presented in Table 1. The data charting form is adapted from prior work [25].

**Table 1:** Data charting form.

Charting information	Explanation (if applicable)
Authors	—^a^
Year of publication	—
Country of origin	Where the study was conducted
Population type	Healthy, at-risk, clinically diagnosed
Population demographic	Age or gender or race
Sample size	—
Intervention	Yes or no
If yes, intervention type	—
If yes, duration of intervention	—
Type of wearable	That is a wrist-worn wearable, smartphone, and mobile application.
Modifiable risk factor	That is (a) physiological risk factor, (b) lifestyle risk factor, (c) or both
Modifiable risk factor measure	risk factor type (ie, physical activity)
Unit of measurement	Measure operationalization (ie, step count per day)
Sampling method	That is (a) ecological momentary assessment or self-report; (b) passive sensing.
Measure sampling frequency	—
Overall measurement duration	—
Number of total risk factors	—

^a^Not applicable.

### Stages 6 and 7: Data analysis and presentation of results

Following the data charting process outlined in Table 1, the gathered data will be synthesized and summarized in descriptive tables and figures. According to our first research question, we will categorize the modifiable risk factors per each study. Next, we will aggregate the overall prevalence of each modifiable risk factor and the proportion of studies that measure single or multiple modifiable risk factors. Based on our second question, we will describe the measures used to operationalize the most prevalent factors, and we will overview the overlap (vs discrepancies) in measurement, that is, how consistently risk factors are measured across studies. The findings will be summarized and communicated through tables and figures. Relevant findings will be communicated to diverse stakeholders, such as digital metabolic health researchers, healthcare professionals, digital health interest groups, metabolic disease patient organizations, and health insurance providers, through presentations and workshops.

### Ethical Considerations

No ethics approval is required for this study as it does not involve conducting trials or collecting primary data.

## Results

A structured search strategy was developed to summarize the landscape of modifiable risk factors for metabolic health and their measurements in the context of wearable and smartphone research. As of the submission of this protocol in October 2024, no data collection has begun. Data collection is scheduled to begin in November 2024, with data analysis set to start in the first quarter of 2025. The results are anticipated to be submitted for peer-reviewed publication as a scoping review by the second quarter of 2025 and will be disseminated through publication in relevant journals and presentations at conferences.

## Discussion

### Anticipated Findings

Prior research [8,29,32] has demonstrated that multiple modifiable risk factors jointly contribute to metabolic disease outcomes. In parallel, a growing number of studies are leveraging wearables and smartphones to track metabolic diseases in daily life (for example, [33,34]). However, it remains unclear whether existing studies examine multiple modifiable risk factors simultaneously or focus on a single factor in isolation. Our results are expected to reveal whether, and the degree to which, studies examine a range of modifiable risk factors in parallel, thereby offering a comprehensive approach, or if they predominantly isolate a single factor, which would suggest a gap in integrating broader determinants of metabolic diseases. Anticipated findings may indicate that certain risk factors, such as physical activity or diet, are more commonly tracked, while others, like stress or sleep, might be underrepresented, thereby revealing potential gaps in the current research focus. Our results could also highlight a strong consistency in the way risk factors are measured (eg, uniform use of validated metrics across studies) or substantial variation in measurement approaches. In the latter case, this inconsistency could point to challenges in comparing or integrating findings across different studies, thereby underscoring the need for standardization in risk factor measurement using wearables and smartphones.

The scoping review is expected to extend the existing literature in several ways. First, Lee et al [20], have begun to outline the characteristics of wearable devices to track cardiometabolic outcomes, but have not examined the degree to which studies examine different risk factors collectively, nor the consistency in measurement across studies. Our review aims to fill these gaps by systematically assessing the extent to which different risk factors are measured together (rather than in isolation), and by evaluating the consistency in measurement across studies. Previous reviews have primarily focused on the application of wearables or digital health technologies within the context of a single disease such as obesity or diabetes [23,35-37], or a single risk factor (eg, nutrition, physical activity, or stress) [14,38-41]. In contrast, our scoping review aims to provide a more comprehensive risk-factor perspective in a broader metabolic health framework.

To the best of our knowledge, this protocol is among the first systematic attempts to outline the current landscape of modifiable risk factors in digital metabolic health research, with a particular focus on the consistency of risk factor measurement across studies. We expect this research to contribute to existing knowledge by systematically scoping the variability in wearable and smartphone-based measurements, thus paving the way for improved measurement standards and comparability across studies. This scoping review will have several limitations, such as only including literature restricted in English, and quantitative empirical studies, which may omit more recent qualitative or industry developments. Our results will be specific to body-worn wearables costing up to €500 (US $570), a price point that may still be considered expensive. As such, some wearables included in our analysis may still be financially out of reach for many, reflecting a potential barrier to accessibility. We will also exclude other potentially relevant devices that are not worn on the body such as breath analyzers. Furthermore, there is often no consensus on the definition of metabolic health in the literature [42], which may lead to varied interpretations of metabolic risk factors. However, drawing from previous studies, we have compiled an extensive list of modifiable risk factors, encompassing both lifestyle behaviors and physiological indicators [43-50]. Future research may compare how different risk factors, and measurement approaches, predict metabolic disease outcomes.

### Conclusion

Overall, this scoping review aims to synthesize existing research on how wearable and smartphone-based studies track modifiable risk factors in digital metabolic health. This work may potentially motivate the development of measurement reporting standards, thereby improving the consistency and applicability of measurements in digital metabolic health studies.

## Appendix

Multimedia Appendix 1 Search strategy for modifiable risk factors in metabolic health studies.

## Abbreviations

JBI: Joanna Briggs Institute
PCC: population, concept, and context
PRISMA-ScR: Preferred Reporting Items for Systematic Reviews and Meta-Analyses Extension for Scoping Reviews
