# Supplementary material for: Wearables and Smartphones for Tracking Modifiable Risk Factors in Metabolic Health: Protocol for a Scoping Review
Source: JMIR Res Protoc. 2024 Nov 28;13:e59539. doi: 10.2196/59539 (PMC11638682; doi:10.2196/59539)
Supplement: Multimedia Appendix 1 [file resprot_v13i1e59539_app1.docx]

Table S1. Search Strategy/Queries

| **Terms** | **Number** | **Syntax** |
| --- | --- | --- |
|  |  |  |
| Metabolic health | #1 | "metabolic syndrome ”OR "metabolic syndromes” OR "insulin resistance syndrome" OR "insulin resistance" OR “metabolic health" OR “metabolic disease" OR “metabolic diseases" OR “syndrome X" OR “Cardiometabolic health" OR “Cardiometabolic syndrome" OR “Type 2 Diabetes” OR “Type 2 Diabetes Mellitus” OR “Cardiovascular Diseases” OR “Cardiovascular Disease” |
| [Wearables, and](https://www.ncbi.nlm.nih.gov/pmc/articles/PMC9641516/) smartphones | #2 | "digital biomarker” OR "digital biomarkers"OR "wearable” OR “wearable electronic devices” OR "wearables" OR “apple watch” OR “fitbit” OR “garmin” OR “whoop” OR “oura ring” OR “jawbone” OR “mio” OR “misfit” OR “polar” OR “Samsung” OR “under armour” OE “withings” OR “xiaomi” OR "smartphone" OR "mobilephone" OR "app" OR "apps" OR "application" OR "applications" OR "implantable" OR “mHealth” OR “ecological momentary assessment“ OR “ EMA” OR “remote monitoring” OR “fitness tracker” OR “fitness trackers” OR “smartwatch” OR “smartwatches” OR “smart ring” OR “smart rings” OR “heart rate monitor" OR “hear rate monitors" OR “glucose monitor" OR “glucose monitors” OR “cell phone” OR “cellular phone” OR “mobile phone” OR (“activity” AND (“monitor” OR “sensor” OR “sensing” OR “device” OR “tech”)) OR (“motion” AND (“monitor” OR “sensor” OR “sensing” OR “device” OR “tech”)) OR “acceleromet*” OR “sensewear” OR “omron” OR “actigraph” |
| **Modifiable risk factors** | #3 |  |
| Physiological markers | #3.1 | "weight loss" OR "weight gain" OR weightloss OR "weight change" OR "weight reduction" OR "weight management" OR "body mass index" OR bmi OR adipo* OR "body composition*" OR "body size*" OR "weight measur*" OR "subcutaneous fat*" OR "abdominal fat*" OR “obesity” OR “overweight” OR “Metabolic Syndrome” OR “obes*” OR “Adiposity” OR “Adipos*” OR “Blood Glucose” OR “Glucose Tolerance Test” OR “Resistance, Insulin” OR “insulin sensitive*” OR “hyperinsulinaemia” OR “glucose metabolism” OR “glucose transport” OR “hyperglycemia” OR “Diabetes Mellitus”  OR “Cholesterol” OR “Triglycerides” OR “Dyslipidemia” OR “Epicholesterol” OR “HDL” OR “LDL” OR “Triglyceride*“ OR “Hyperlipidem*” OR “Lipidem*” OR “Hypertension” OR “Triglycerides” OR “Hypertens*” OR “Prehypertens*” OR “Systolic” OR “Diastolic” |
| Lifestyle behavior | #3.2 |  |
| Nutrition | #3.2.1 | ("Nutrition Assessment” OR "Nutrition Surveys"  OR "Diet” OR “food” OR “foods” OR “Nutrition" OR  "Eating" OR "Feeding Behavior" OR “feeding” OR "dietary behavior" OR "dietary” OR “food intake" OR “nutrient” OR “nutrients”) |
| Physical activity | #3.2.2 | (“exercise” OR "healthy Lifestyle" OR "physical activit*" OR "physical Fitness" OR "health behavio*" OR "fitness" OR "bicycle" OR "stair climb" OR "strength training" OR "resistance training" OR "active travel" OR "active transport" OR “acceleromet*” OR “walking” OR “sedentary” OR "physical inactivit*" OR “sitting” OR "screen time" OR "screen use" OR "social media" OR “sport” OR “sports” OR “leisure” OR “leisure activ*” OR “run*” OR “training” or “trainings” OR “walk*” |
| Stress | #3.2.3 | “mental health” OR “mental disorder” OR “psychological distress” OR “mental distress” OR “stress” OR “distress” |
| Sleep | #3.2.4 | “sleep*” |
| Substance use | #3.2.5 | “substance” OR “alcohol” OR “drug” OR “opioid” OR “tobacco”) AND (“abuse” OR “misuse” OR “withdrawal” OR “abstinence” OR “addict* OR “craving” OR “dependency” OR “illegal” OR “illicit” OR “overdose” OR “prevention” OR “user” OR “intake” OR “recreational” OR “recovery” OR “smoke” OR “smok*” OR "cessation" OR "reduction" OR "use" OR "quit" OR "stop" OR "reduct*" OR "reduce" OR "dehabituat*" OR "abstinen*")) OR "vaping" OR "e-cigarettes" |
| **Study population** | #4 | (“adult*” OR "middle aged" OR "young adult*" OR "grown-up*"")  NOT  (“adolescen*” OR “infant*” OR “child*” OR "school-aged" OR “preschool”) |
| **Publication date** | #5 | “2019/01/01”[Date - Publication]: “2024/XX/XX”[Date - Publication] |
| **Search query** | #1 AND #3 AND (#3.1 OR #3.2.1 OR #3.2.2 OR #3.2.3 OR #3.2.4 OR #3.2.5) AND #4 AND #5 |  |

Table S2. Sample Database search strategy – PubMed

Search performed on August 15^th^ 2024

| **Database search** | **Search results** |
| --- | --- |
| ("metabolic syndrome"[Title] OR "metabolic syndromes"[Title] OR "insulin resistance syndrome"[Title] OR "insulin resistance"[Title] OR "metabolic health"[Title] OR "metabolic disease"[Title] OR "metabolic diseases"[Title] OR “metabolic disorder” [Title] OR "syndrome X"[Title] OR "Cardiometabolic health"[Title] OR "Cardiometabolic syndrome"[Title] OR "Type 2 Diabetes"[Title] OR "Type 2 Diabetes Mellitus"[Title] OR "Cardiovascular Diseases"[Title] OR "Cardiovascular Disease"[Title]) | 191,300 |
| *AND* |  |
| ("digital biomarker"[Title/Abstract] OR "digital biomarkers"[Title/Abstract] OR "wearable"[Title/Abstract] OR "wearable electronic devices"[Title/Abstract] OR "wearables"[Title/Abstract] OR "apple watch"[Title/Abstract] OR "fitbit"[Title/Abstract] OR "garmin"[Title/Abstract] OR "whoop"[Title/Abstract] OR "oura ring"[Title/Abstract] OR "jawbone"[Title/Abstract] OR "mio"[Title/Abstract] OR "misfit"[Title/Abstract] OR "polar"[Title/Abstract] OR "Samsung"[Title/Abstract] OR ("under"[Title/Abstract] AND "armour"[Title/Abstract]) OR "withings"[Title/Abstract] OR "xiaomi"[Title/Abstract] OR "smartphon*"[Title/Abstract] OR "mobilephon*"[Title/Abstract] OR "app"[Title/Abstract] OR "apps"[Title/Abstract] OR "application"[Title/Abstract] OR "applications"[Title/Abstract] OR "implantable"[Title/Abstract] OR "mHealth"[Title/Abstract] OR "ecological momentary assessment"[Title/Abstract] OR "EMA"[Title/Abstract] OR "remote monitoring"[Title/Abstract] OR "fitness tracker"[Title/Abstract] OR "fitness trackers"[Title/Abstract] OR "smartwatch"[Title/Abstract] OR "smartwatches"[Title/Abstract] OR "smart ring"[Title/Abstract] OR "smart rings"[Title/Abstract] OR "heart rate monitor"[Title/Abstract] OR "heart rate monitors"[Title/Abstract] OR "glucose monitor"[Title/Abstract] OR "glucose monitors"[Title/Abstract] OR "cell phone"[Title/Abstract] OR "cellular phone"[Title/Abstract] OR "mobile phone"[Title/Abstract] OR ("activity"[Title/Abstract] AND ("monitor"[Title/Abstract] OR "sensor"[Title/Abstract] OR "sensing"[Title/Abstract] OR "device"[Title/Abstract] OR "tech"[Title/Abstract])) OR ("motion"[Title/Abstract] AND ("monitor"[Title/Abstract] OR "sensor"[Title/Abstract] OR "sensing"[Title/Abstract] OR "device"[Title/Abstract] OR "tech"[Title/Abstract])) OR "acceleromet*"[Title/Abstract] OR "sensewear"[Title/Abstract] OR "Omron"[Title/Abstract] OR "Actigraph"[Title/Abstract])  *AND* | 2,077,637 |
| (("weight loss"[Title/Abstract] OR "weight gain"[Title/Abstract] OR "weightloss"[Title/Abstract] OR "weight change"[Title/Abstract] OR "weight reduction"[Title/Abstract] OR "weight management"[Title/Abstract] OR "body mass index"[Title/Abstract] OR "bmi"[Title/Abstract] OR "adipo*"[Title/Abstract] OR "body composition*"[Title/Abstract] OR "body size*"[Title/Abstract] OR "weight measur*"[Title/Abstract] OR "subcutaneous fat*"[Title/Abstract] OR "abdominal fat*"[Title/Abstract] OR "obesity"[Title/Abstract] OR "overweight"[Title/Abstract] OR "Metabolic Syndrome"[Title/Abstract] OR "obes*"[Title/Abstract] OR "Adiposity"[Title/Abstract] OR "Adipos*"[Title/Abstract] OR "Blood Glucose"[Title/Abstract] OR "Glucose Tolerance Test"[Title/Abstract] OR "Insulin resistanc*"[Title/Abstract] OR "insulin sensitiv*"[Title/Abstract] OR "hyperinsulinaemia"[Title/Abstract] OR "glucose metabolism"[Title/Abstract] OR "glucose transport"[Title/Abstract] OR "hyperglycemia"[Title/Abstract] OR "Diabetes Mellitus"[Title/Abstract] OR "Cholesterol"[Title/Abstract] OR "Triglycerides"[Title/Abstract] OR "Dyslipidemia"[Title/Abstract] OR "Epicholesterol"[Title/Abstract] OR "HDL"[Title/Abstract] OR "LDL"[Title/Abstract] OR "Triglyceride*"[Title/Abstract] OR "Hyperlipidem*"[Title/Abstract] OR "Lipidem*"[Title/Abstract] OR "Hypertension"[Title/Abstract] OR "Hypertens*"[Title/Abstract] OR "Prehypertens*"[Title/Abstract] OR "Systolic"[Title/Abstract] OR "Diastolic"[Title/Abstract]) | 2,168,132 |
| *OR* |  |
| ("Nutrition Assessment"[Title/Abstract] OR "Nutrition Surveys"[Title/Abstract] OR "Diet"[Title/Abstract] OR "food"[Title/Abstract] OR "foods"[Title/Abstract] OR "Nutrition"[Title/Abstract] OR "Eating"[Title/Abstract] OR "Feeding Behavior"[Title/Abstract] OR "feeding"[Title/Abstract] OR "dietary behavior"[Title/Abstract] OR "dietary"[Title/Abstract] OR "food intake"[Title/Abstract] OR "nutrient"[Title/Abstract] OR "nutrients"[Title/Abstract]) | 1,614,614 |
| *OR* |  |
| ("exercise"[Title/Abstract] OR "healthy Lifestyle"[Title/Abstract] OR "physical activit*"[Title/Abstract] OR "physical Fitness"[Title/Abstract] OR "health behavio*"[Title/Abstract] OR "fitness"[Title/Abstract] OR "bicycle"[Title/Abstract] OR "stair climb"[Title/Abstract] OR "strength training"[Title/Abstract] OR "resistance training"[Title/Abstract] OR "active travel"[Title/Abstract] OR "active transport"[Title/Abstract] OR "walking"[Title/Abstract] OR "sedentary"[Title/Abstract] OR "physical inactivit*"[Title/Abstract] OR "sitting"[Title/Abstract] OR "screen time"[Title/Abstract] OR "screen use"[Title/Abstract] OR "social media"[Title/Abstract] OR "sport"[Title/Abstract] OR "sports"[Title/Abstract] OR "leisure"[Title/Abstract] OR "leisure activ*"[Title/Abstract] OR "run"[Title/Abstract] OR "runing"[Title/Abstract] OR "training"[Title/Abstract] OR "trainings"[Title/Abstract] OR "walk"[Title/Abstract]) | 1,451,875 |
| *OR* |  |
| ("mental health"[Title/Abstract] OR "mental disorder"[Title/Abstract] OR "psychological distress"[Title/Abstract] OR "mental distress"[Title/Abstract] OR "stress"[Title/Abstract] OR "distress"[Title/Abstract]) | 1,416,336 |
| *OR* |  |
| ("sleep*"[Title/Abstract]) | 252,649 |
| *OR* |  |
| ((("substance"[Title/Abstract] OR "alcohol*"[Title/Abstract] OR "drug"[Title/Abstract] OR "opioid"[Title/Abstract] OR "tobacco"[Title/Abstract]) AND ("abuse"[Title/Abstract] OR "misuse"[Title/Abstract] OR "withdrawal"[Title/Abstract] OR "abstinence"[Title/Abstract] OR "addict*"[Title/Abstract] OR "craving"[Title/Abstract] OR "dependency"[Title/Abstract] OR "illegal"[Title/Abstract] OR "illicit"[Title/Abstract] OR "overdose"[Title/Abstract] OR "prevention"[Title/Abstract] OR "user"[Title/Abstract] OR "intake"[Title/Abstract] OR "recreational"[Title/Abstract] OR "recovery"[Title/Abstract] OR "smok*"[Title/Abstract] OR "cessation"[Title/Abstract] OR "reduction"[Title/Abstract] OR "use"[Title/Abstract] OR "quit"[Title/Abstract] OR "stop"[Title/Abstract] OR "reduct*"[Title/Abstract] OR "reduce"[Title/Abstract] OR "dehabituat*"[Title/Abstract] OR "abstinen*"[Title/Abstract])) OR "vaping"[Title/Abstract] OR "e-cigarettes"[Title/Abstract] OR "e-cigarette"[Title/Abstract])) | 962,940 |
|  | **Total:** 4,455 |
| *AND* |  |
| (alladult[Filter] OR youngadult[Filter] OR adult[Filter] OR middleagedaged[Filter] OR middleaged[Filter] OR aged[Filter] OR 80andover[Filter]) | 1,463 |
| *AND* |  |
| ("2019/01/01"[Date - Publication] : "2024/12/31"[Date - Publication]) | 642 |
| *AND* |  |
| "English"[la] | **Final search results: 633** |
